# Continuous sensing and quantification of body motion in infants: A systematic review

**DOI:** 10.1016/j.heliyon.2023.e18234

**Published:** 2023-07-13

**Authors:** Zheng Peng, Deedee Kommers, Rong-Hao Liang, Xi Long, Ward Cottaar, Hendrik Niemarkt, Peter Andriessen, Carola van Pul

**Affiliations:** a Department of Applied Physics, Eindhoven University of Technology, Eindhoven, the Netherlands; b Department of Clinical Physics, Máxima Medical Centre, Veldhoven, the Netherlands; c Department of Electrical Engineering, Eindhoven University of Technology, Eindhoven, the Netherlands; d Philips Research, Eindhoven, the Netherlands; e Department of Industrial Design, Eindhoven University of Technology, Eindhoven, the Netherlands; f Department of Neonatology, Máxima Medical Centre, Veldhoven, the Netherlands

**Keywords:** Infant, Motion monitoring, Sensing technology

## Abstract

Abnormal body motion in infants may be associated with neurodevelopmental delay or critical illness. In contrast to continuous patient monitoring of the basic vitals, the body motion of infants is only determined by discrete periodic clinical observations of caregivers, leaving the infants unattended for observation for a longer time. One step to fill this gap is to introduce and compare different sensing technologies that are suitable for continuous infant body motion quantification. Therefore, we conducted this systematic review for infant body motion quantification based on the PRISMA method (Preferred Reporting Items for Systematic Reviews and Meta-Analyses). In this systematic review, we introduce and compare several sensing technologies with motion quantification in different clinical applications. We discuss the pros and cons of each sensing technology for motion quantification. Additionally, we highlight the clinical value and prospects of infant motion monitoring. Finally, we provide suggestions with specific needs in clinical practice, which can be referred by clinical users for their implementation. Our findings suggest that motion quantification can improve the performance of vital sign monitoring, and can provide clinical value to the diagnosis of complications in infants.

## 1 Introduction

To monitor the health status of hospitalized infants closely, their heart rate, respiration rate, and oxygen saturation are monitored continuously. This is common practice already for decades. However, in commercially available patient monitors, there is no available sensor or technology applied to determine a patient's body motion. Although body motion is thought to be a relevant indicator of a patient's clinical status, body motion is only determined by clinical observations from nurses and clinicians, and is not monitored continuously.

In the last years, infant body motion quantification has gained increasing interest in research, especially in the neonatal intensive care unit (NICU) and neonatal ward. One reason for this is that vital sign monitoring is prone to motion artifacts, resulting in false alarms in the NICU and neonatal ward [1] and the detection of motion artifacts can thus be used to compensate and correct the monitored vital signs to improve the monitoring performance. This is particularly important for non-contact vital sign monitoring which has gained popularity in neonates but still faces a big challenge with motion artifacts, as reviewed by Maurya et al. [2]. The second reason is that body motion patterns themselves are associated with the neurodevelopment of infants and deviating patterns may be indicative of suboptimal development or even pathological conditions. For example, the motion pattern of more mature infants is characterized by gross motion that lasts around 5 s or longer, while prematurity is characterized by more frequent, briefer, and finer motion [3]. In addition, the motion signal (e.g. measured with actigraphy) may also be used to differentiate between (ab)normal sleep development and sleep stages in infants [4]. Furthermore, the assessment of spontaneous general motion can be a useful predictor of motion-motor impairments in high-risk infants [5] and lack of motion is one of the clinical signs before the onset of sepsis [6]. Therefore, continuous motion quantification with sensing technologies is an important need in care units for infants.

Currently, although there are observational scales for quantifying motion in infants, these are not routinely used since they are labor-intensive and require specific training. In clinical practice, the motion patterns are only qualitatively interpreted during intermittent observations from caregivers. Whilst clinical observations are considered the gold standard, these are suboptimal because of their discontinuous nature and inter-rater subjectivity, which may lead to missing clinically important events. Automated motion measurement may provide continuous and additional information that may be useful for supporting diagnostic decision-making. In 2015, Marcroft et al. [5] reported in a mini-review showing that motion recognition technology can be an important tool to predict neurological and motor impairments in high-risk infants, enhancing the understanding of the development of the nervous system in infants at high risk of motor impairment. In their review, indirect sensing methods based on camera systems and direct sensing methods based on wearable motion sensors are included, focusing on capturing abnormal motion patterns, particularly relevant limb motion in infants [5]. As a further step, Raghuram et al. [7] reviewed the diagnostic performance of motion recognition technologies in motor impairments prediction in high-risk infants, indicating the need for further development of the technology and more validation studies. Additionally, two other reviews in 2015 summarized direct sensing technologies (e.g. wearable sensors) that have been investigated to monitor the body motion of infants [8] and discussed sensor types and the technology implementation possibilities [9]. However, these reviews do not address the crucial aspect of quantifying the motion into a vital sign that can be continuously displayed, which can be essential for real-time infant monitoring in clinical practice. In recent years, several new studies have been published on quantification possibilities for motion monitoring in infants.

The main aim of this study is to review and compare the sensing technologies for continuous body motion quantification in infants. Researchers and clinical practitioners who aim to perform a study to contribute to further validation of any type of sensing technology for continuous infant body motion quantification may use this as a reference for choosing the appropriate sensing technology depending on the foreseen clinical application and environment. To our best knowledge, this review is the first study that compares sensing technologies of continuous body motion quantification for infants. This review is organized similarly to the systematic review on non-contact breathing rate monitoring in newborns by Maurya et al. [2]. In section 2, the systematic review method is addressed. In section 3, the results of the systematic review are presented and discussed based on the different sensing technologies for motion quantification in infants. Special focus will be put on methods for motion quantification. Section 4 will discuss the aims of the clinical application of motion quantification. The challenges and limitations of each motion-sensing technology as well as future directions are discussed and outlined. Section 5 gives the conclusion for motion quantification in infants.

## 2 Systematic review method

**Fig. 1:**
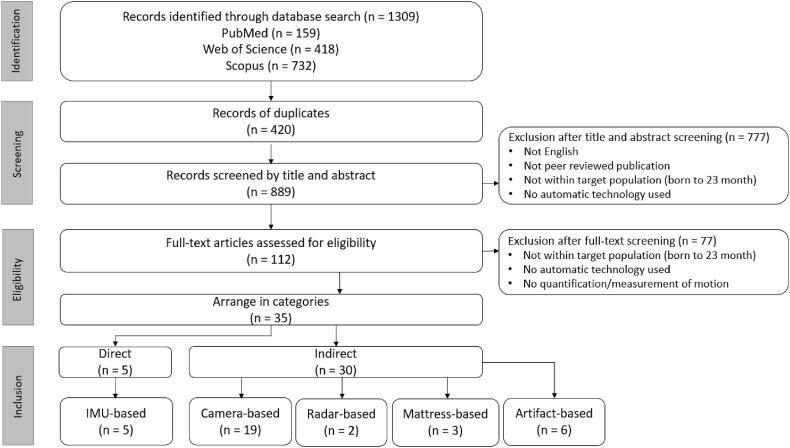
PRISMA flow diagram of literature search and screening process.

This systematic review was performed according to the PRISMA method (Preferred Reporting Items for Systematic Reviews and Meta-Analyses). We first applied our search strategy to three publication databases: PubMed, Web of Science, and Scopus. We targeted relevant papers within the recent 10 years (2012–2022). The search strategy included four aspects that all had to be present for a paper to be included: infant, motion, continuous, and monitoring. The search terms and used synonyms are shown in Table 1, and the flow of retrieved hits and included papers can be seen in the flow diagram (Fig. 1). All identified 1309 papers from the three databases were deduplicated, resulting in 889 papers left for the first round of screening where two authors included papers based on title and abstract. When the two authors disagreed on inclusions, a third author would make the final decision. In this first round of screening, the first three criteria (infant, motion, and continuous) for inclusion needed to be met. This led to a total of 112 identified papers for the second round of screening which assessed eligibility based on the full text. In this second round of inclusion, eligibility was assessed additionally based on a (clear) description of motion quantification. Furthermore, only the peer-reviewed English papers where the subjects were below 23 months of age, and contained automatic sensing technology were included. Papers that used quantified motion as the primary objective (e.g. for continuous monitoring) and papers that quantified motion as an intermediate step for the improvement of other clinical applications were both included.

**Table 1:** Search terms used in publication databases.

Infant	Infant OR Neonat* OR Newborn
AND	
Motion	Movement OR Motion OR Moving OR Position OR Pose OR “Motor behavior”
AND	
Continuous	Continuous OR Realtime OR “Real time”
AND	
Monitoring	Monitor* OR Detect* OR Quantif* OR Estimat*

* is the truncation wildcard.

**Fig. 2:**
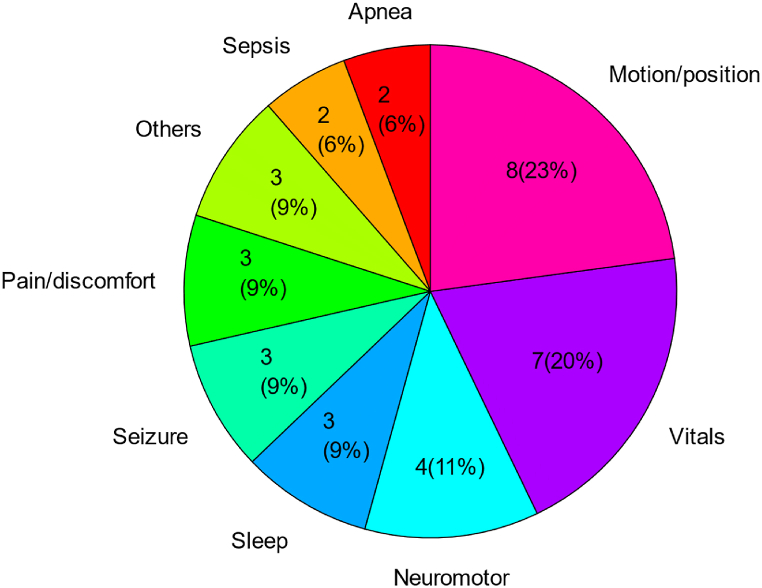
Publications in different categories of applications.

Finally, thirty five papers were included in the analysis of this review. Among these thirty five papers, as shown in Fig. 2, eight papers (23%) focused on motion/position monitoring as the primary goal. Seven papers (20%) quantified motion as an intermediate step for further improvement of vital sign monitoring (e.g. heart rate, respiration rate). The rest of the papers used quantified motion to detect deviating patterns with pathological conditions including neuromotor pathology (11%), sleep (9%), seizure (9%), pain/discomfort (9%), sepsis (6%), and apnea (6%).

## 3 Methods for motion sensing and quantification

The sensing technologies of continuous body motion quantification can be categorized into direct sensing and indirect sensing. In the case of direct sensing, the hardware (e.g. sensor) is directly attached to the subject to monitor the body motion. While in the case of indirect sensing, the motion is captured by hardware placed in the monitoring environment or derived from other (directly) monitored signals.

### 3.1 Direct sensing technology

**Fig. 3:**
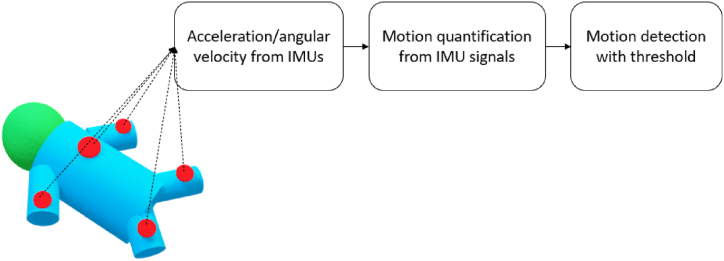
The block diagram of direct sensing technology for motion detection.

For direct sensing of body motion, the inertial measurement unit (IMU) including accelerometers and gyroscopes has been used in infants in several studies [4,[10], [11], [12], [13]]. A 3-axis accelerometer can detect linear acceleration along each axis, whereas gyroscopes can detect angular velocity. For details on this sensing technology, we refer to the book by Tamura and Chen [14]. Fig. 3 illustrates the general block diagram of direct sensing technology for motion detection. Table 2 shows the included papers using direct sensing technology, summarizing the patient population, the type of sensing technology, the placement, the aim of motion quantification, and the quantification method used.

**Table 2:** Studies for neonatal motion quantification using direct sensing technology. The order is sorted by the aim of motion quantification, and year.

Author (Refer)	Year	Subject size (P, I)	Sensing technology	Product model	Placement	Aim of motion quantification	Signal processing method of motion quantification
Chung et al. [10]	2020	P = 35 I = 15	Accelerometer & gyroscope	BMI160	Chest, using an epidermal sensor [15]	Motion/position monitoring: Recognize body positions during different activities.	Low-pass filtering, Derived rotation angles
Jeong et al. [13]	2022	I = 2	Accelerometer & gyroscope	BMI160	Multiple limb and head/torso positions, using silicone elastomer with medical silicone adhesives	Neuromotor pathology: Estimate pose and orientation for 3D motion reconstruction	Unified robot description format
Lan et al. [12]	2018	P = 65	Accelerometer	wGT3x-BT	Left and right legs, using a bandage	Sleep: Transform motion counts into sleep stage estimates	Activity is processed based on the method from Sadeh et al. [16]
Schoch et al. [4]	2019	I = 50	Accelerometer	GENEactiv	Left ankle, using a sock or paper strap to position	Sleep: Transform motion counts into sleep estimates	Squared sum of three axes
Raj et al. [11]	2018	P = 10	Accelerometer	LIS2HH12	Sternum or abdomen; using skin adhesive tape	Vitals: Reject unreliable period with motion artifact for respiration estimation	Squared sum of three axes, adaptive filter

P – Preterm infants; I – Infants.

Quantification of actigraphy (motion) signals has been used for a long time in physical behavior and sleep assessment studies, also in infants [[17], [18], [19]]. Sleep assessment methods are usually based on the quantification of raw activity by summation of the accelerometer signals along three axes and often include derived features per time window (e.g. number of motion bursts) that are used to build a function or model that relates activity to sleep stage. Lan et al. [12] assessed the sleep variables of 65 infants such as total sleep time and frequency of wake bouts by converting the quantified limb motion into motion counts with a previously validated algorithm [16]. This motion quantification algorithm for sleep analysis was further compared and optimized by Schoch et al. [4] who minimized the discrepancies in sleep estimates using motion signals between different quantification algorithms, achieving less bias and better agreement (96%–97%) with sleep diary in sleep-wake analysis.

In other applications of continuous motion monitoring, similar summations of raw accelerometer signals can also be used as a simple quantification method for motion. Additionally, IMUs can be used to estimate vital signs such as respiration rate by detecting the periodicity in the motion. The amplitude of the quantified motion (squared sum of the IMU signals over all axis) can be used to detect gross motion (motion artifact) as well. Raj et al. [11] designed a wearable respiratory rate device tested on 10 neonates using a 3-axis accelerometer. In this study, the device was attached to the sternum or abdomen of the infant with soft skin adhesive tape. The accelerometer data were fused to first detect the existence of motion artifacts and then to detect peaks and valleys specific to respiration. This device was found to have a correlation coefficient of 0.97 with the reference respiration values. The quantified motion was not only used as a motion artifact detector to identify the unreliable period of respiration estimation but also could be directly used to quantify infant gross motion. Jeong et al. [13] used 10 miniaturized accelerometers for body motion quantification to study neuromotor motion patterns. On top of this, they also derive vital signs from the accelerometers including respiration and heart rate in infants. Their whole sensor system is small and because of the strategic placement locations (bilaterally on shoulders, wrists, hips, ankles, and xiphoid process, forehead) of IMUs, the system can quantitatively assess motion across the full range of spatiotemporal scales, enabling to reconstruct the continuous 3D motion of infants. Chung et al. [10] designed a soft adhesive patch, also described as an epidermal electronic system for physiological monitoring in infants. Compared to the adhesive electrodes commonly used in the NICU and NMCU, this system reduced forces on the skin of infants [15]. Furthermore, apart from monitoring the standard physiological signals including heart rate, respiration rate, blood oxygenation (SpO2), and temperature, this system also allows for new monitoring possibilities: 1) motion and body orientation, 2) acoustic signatures associated with cardiac activity, 3) vocal biomarkers with tonality and temporal characteristics of crying, and 4) quantified pulse-wave dynamics as a surrogate for systolic blood pressure. Among them, body orientation detection and crying analysis are enabled due to IMU-based motion quantification. Specifically, the suggested body position of infants during kangaroo care (a period of parent-infant skin-to-skin contact during which an infant is usually held upright on the parent's chest) can be continuously monitored by the IMU attached to the chest and is distinct from other activities. The high-frequency chest vibration of crying can also be recorded by the accelerometer for crying analysis. Importantly, this recording method is naturally unaffected by the ambient noise which confounded conventional recording methods like microphones.

IMU-based direct sensing technology offers the advantage of obtaining accurate and continuous motion signals and estimating vital signs, and they are likely to function well during caregiving moments. However, the primary limitation is that direct contact with the sensitive skin of infants increases the risk of infection if the sensor is not adequately sanitized [20], as well as the possibility of skin damage. Although efforts to develop easy-to-use and skin-safe adhesive sensors, such as those created by Jeong et al. [13] and Chung et al. [10], are ongoing, more clinical validations involving a sufficient number of infants are still required. Additionally, the adhesion of sensors necessitates specific manufacturing equipment and expertise, making it currently unavailable commercially. As a result, numerous studies on motion quantification focused on indirect sensing technologies, such as those utilizing cameras and mattresses [10,21].

### 3.2 Indirect sensing technology

Motion quantification using indirect sensing technology can be divided into four categories: camera-based, radar-based, mattress-based, and signal-reusing technologies. The former three technologies use the additional hardware set in the monitoring environment while the last (signal-reusing) technology derives the motion signal from existing monitored physiological signals. Table 3 shows the studies for neonatal motion quantification using different indirect sensing technologies in multiple applications.

**Table 3:** Studies for neonatal motion quantification using indirect sensing technology. The order is sorted by the aim of motion quantification, and year for each sensing technology.

Author (Refer)	Year	Subject size	Sensing technology	Product model	Placement	Aim of motion quantification	Method of motion quantification
Ossmy et al. [21]	2020	I = 1	RGB Camera	Not mentioned	Overhead camera view	Motion/position monitoring: Generate the moving path of the infant	Changes in feet location detected by convolutional pose machine
Zhao et al. [22]	2020	I = 1	RGB Camera	OV5642	On the ceiling of the incubator	Motion/position monitoring: Trigger alarm system to notify caregivers	Background subtraction
Peng et al. [23]	2022	I = 18	RGB/Thermal Camera	UI-3860LE-C-HQ/FLIR Lepton5.5	On top of the incubator or bed	Motion/position monitoring: Motion detection	Background subtraction, optical flow, ORB
Mazzarella et al. [24]	2020	I = 14	RGB Camera	10-camera Vicon motion capture system	In front of infants	Neuromotor pathology: Extract features from quantified motion signals to detect perinatal stroke and cerebral palsy	Changes in 3D coordinates of markers
Malik et al. [25]	2020	I = 2	RGB Camera	Not mentioned	Bedside	Neuromotor pathology: Magnify motion signal to detect tremors	Convolutional encoder-manipulator-decoder network
Wu et al. [26]	2021	I = 59	RGB/RGBD Camera	Kinect	On top of the bed	Neuromotor pathology: Extract features from identified 14-keypoint motion sequence to predict cerebral palsy	Changes in coordinates of 14 keypoints detected by PifPaf
Zamzmi et al. [27]	2016	I = 18	RGB Camera	Not mentioned	On top of the bed	Pain/discomfort detection: Extract a feature from a quantified motion signal to assess pain	Background subtraction
Sun et al. [28]	2019	P = 11	RGB Camera	uEye UI-222x	On the ceiling of the incubator	Pain/discomfort detection: Extract features from quantified motion signals to detect discomfort	Optical flow
Sun et al. [29]	2021	I = 24	RGB Camera	Xacti VPC-FH1BK	On top of the bed (only face view)	Pain/discomfort detection: Quantified motion signals combined with RGB images as inputs of neural network for discomfort detection	Optical flow
Ferrari et al. [30]	2010	I = 1	RGB Camera	Not mentioned	In front of the bed	Seizure detection: Identify periodicity of motion to detect clonic seizures	Background subtraction
Pisani et al. [31]	2014	I = 12	RGB Camera	Not mentioned	In front of bed	Seizure detection: Identify periodicity of motion to detect clonic seizures	Background subtraction
Martin et al. [32]	2022	I = 43	RGB Camera	Cadwell systems	On top of the bed	Seizure detection: Extract power from the quantified motion signal to detect seizure	Optical flow
Mestha et al. [33]	2014	I = 8	RGB Camera	Not mentioned	Bedside	Vitals: Reject unreliable period with motion artifact for pulse rate estimation.	Background subtraction
Rossol et al. [34]	2020	I = 2	RGB (YUV) Camera	Wansview	4–6 feet on top of the bed	Vitals: Extract respiration from the motion signal	Background subtraction
Lorato et al. [35]	2021	P = 5 I = 12	RGB/Thermal Camera	UI-2220SE/FLIR Lepton5.5	Bedside	Vitals: Reject unreliable period with motion artifact for respiration estimation	Background subtraction
Lyra et al. [36]	2022	I = 19	Thermography Camera	VarioCAM HD head 820 S	On top of the bed	Vitals: Reject unreliable period with motion artifact for respiration estimation	Displacement of the bounding box of the head detected by YOLOv4-Tiny
Long et al. [37]	2018	I = 5	IR Camera	Philips Avent uGrow baby monitor	In front of the bed	Others: Detect infant presence and in/out bed motion	3D recursive search
Chaichulee et al. [38]	2019	P = 15	RGB Camera	3-CCD JAI AT-200CL digital video camera (JAI A/S, Denmark)	Inside the incubator through a hole	Others: Quantified motion signals combined with image signals as inputs of neural networks for intervention detection	Optical flow
Andrea et al. [39]	2022	P = 201 I = 501	In-bore Camera	Not mentioned	Inside MRI	Others: Motivate MRI operators to adjust scan protocols accordingly	Framewise displacement
Lee et al. [40]	2020	P = 16 I = 18	Radar	IR-UWB Radar	35 cm orthogonal away from the chest	Vitals: Reject unreliable period with motion artifact for cardiorespiratory monitoring	Power differences
Beltrao et al. [41]	2022	P = 12	Radar	Radar in 24-GHZ ISM band	45–50 cm orthogonal away from the chest	Vitals: Identify and mitigate motion artifacts for respiration estimation	Nonnegative matrix factorization
Joshi et al. [42]	2018	P = 10	Mattress/BSG	electromechanical film sensor (EMFi, Emfitt, Kuopio, Finland)	On top of the regular mattress and covered by the bedsheet	Motion/position monitoring: Gross motion detection Motion Monitoring	Signal instability index from BSG
Aziz et al. [43]	2020	I = 5	Mattress/BSG	LX100:100.100.05 (XSensor Technology Corp)	Between bedding and infant	Motion/position monitoring: Gross motion detection	Displacement of the center of the pressure
Ranta et al. [44]	2021	I = 43	Mattress/BSG	Electromechanical ferroelectric sensor (L-4060SLC, Emfit, Finland)	Not mentioned	Sleep: Extract motion-related features for sleep stage classification	Smoothed root mean square value of BSG
Williamson et al. [45]	2013	P = 6	PPG reusing	Not appliable	Not appliable	Apnea detection: Extract motion-related features for apnea prediction	Low-frequency power in PPG
Zuzarte et al. [46]	2021	P = 10	PPG reusing	Not appliable	Not appliable	Apnea detection: Extract motion-related features for apnea prediction	Low-frequency power in PPG
Zuzarte et al. [3]	2019	P = 18	PPG reusing	Not appliable	Not appliable	Motion/position monitoring: Gross motion detection	Low-frequency power in PPG
Peng et al. [47]	2021	I = 15	ECG, PPG, CI reusing	Not appliable	Not appliable	Motion/position Monitoring: Gross motion detection	Signal instability index and/or Low-frequency power
Cabrera-Quiros et al. [48]	2021	P = 64	ECG reusing	Not appliable	Not appliable	Sepsis detection: Extract motion-related features for sepsis prediction	Signal instability index from ECG
Peng et al. [49]	2022	P = 127	ECG, CI reusing	Not appliable	Not appliable	Sepsis detection: Extract motion-related features for sepsis prediction	Signal instability index and/or Low-frequency power

P – Preterm infants; I – Infants; BSG -- ballistography; PPG -- photoplethysmogram; ECG -- electrocardiogram; CI – chest impedance.

#### 3.2.1 Camera-based

**Fig. 4:**
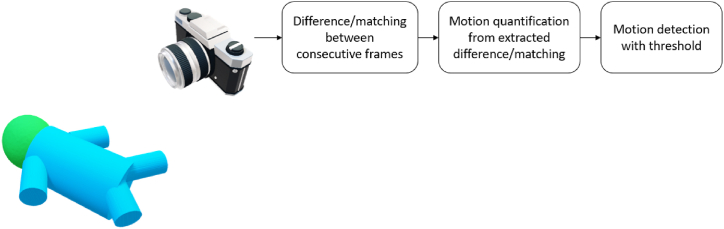
The block diagram of camera-based technology for motion detection.

Camera-based motion detection is a well-known application in surveillance monitoring [50,51]. Conventional camera-based technology detects motion by comparing the difference or matching the blocks between consecutive frames. The block diagram of this technology is shown in Fig. 4. Typical quantification methods include background subtraction [52], optical flow [53,54], and block-matching algorithms [55]. The changing pixels and the displacement of matched blocks in these algorithms can be quantified as motion signals. Since the NICU environments are usually dark, and RGB-based technologies suffer from this low light condition, infrared (IR)/thermal camera-based technologies have drawn attention and have been investigated in the context of motion detection in infants. Peng et al. [23] did a comparison study of video-based methods (both in RGB and IR systems) for motion detection in 18 infants and compared the performance to the manual annotation of fine and gross motion. This study illustrated the feasibility of 4 different motion quantification methods including background subtraction, sparse optical flow, dense optical flow, and oriented FAST and rotated BRIEF (ORB) [56]. Background subtraction method performed well on automatic motion detection in thermal videos and sparse optical flow outperformed in RGB videos, obtaining area under the receiver operating curves (AUCs) of 0.86 and 0.82 respectively in motion detection validated by manual video-based annotation. Also using the background subtraction method, Cabon et al. characterized the motion activities of infants and detect the presence of caregivers with an accuracy of 95% [57]. Zhao et al. [22] developed a platform to notify medical staff when the large-range motions of infants (also can be the fall of an oxygen tube) were detected.

Apart from motion detection, motion quantification with camera setups was also developed in multiple other applications like vital sign estimation in both home-care and hospital-care scenarios. On the one hand, the quantified motion can be used as an artifact detector to find periods in which motion disturbs the monitored metric of interest. On the other hand, vital signs can be estimated from the quantified motion by detecting the periodicity of the motion signal due to the periodic motion generated by breathing and heart beating. Mestha et al. [33] used a camera setup to detect pulse rate from videoplethysmographic signals, using background subtraction based motion detection in the video images to compensate for motion artifacts from each region of interest where the pulse rate is determined. Similarly, to get rid of the interference by gross motion, Lorato et al. [35] developed a gross motion detector based on the background subtraction method to only keep useable video sequences for respiration estimation from videos. Rossol et al. [34] amplified the motion in videos with the Eulerian video magnification algorithm, followed by a motion history image algorithm to quantify motion, and finally used the micromotion and stationarity detection algorithm to estimate respiration from the quantified motion. In the study by Andrea et al. [39], to perform MRI scanning only in low-motion periods, the motion was quantified by framewise displacement and then the visualization of the continuous motion metrics was shown to technicians. In addition to this, more advanced methods such as deep learning models can also be able to detect the motion of an object. For instance, the deep-learning-based YOLOv4-Tiny object detector [58] was applied in the study conducted by Lyra et al. [36] to identify the head and nose of infants. The motion of the head and nose were quantified to discard sections with motion interference in their signal of interest.

Besides, in several papers, camera-based motion quantification was used to identify motion patterns of neuromotor pathology (i.e. cerebral palsy prediction), seizure detection, and pain/discomfort detection. For instance, Mazzarella et al. [24] used a 10-camera Vicon motion capture system to record the 3D coordinates of 8 markers that were placed on the infants. The changes in the coordinates of the markers were quantified as infant motion and then were summarized by several features to detect perinatal stroke and cerebral palsy in infants. To reveal subtle tremors in infants, Malik et al. [25] used a fully convolutional encoder-manipulator-decoder network to magnify motion between frames. To better quantify the motion and estimate the posture of infants, Wu et al. [26] applied a neural network called PifPaf [59] to estimate the coordinates of 14 key points in each frame and thereby extracted features from the changes of coordinates to predict the risk of cerebral palsy in infants, achieving detection accuracy of 91.5%. Further to this, Ossmy et al. [21] developed a computer-vision framework to accelerate research in human development by automatically tracking the body locations of infants based on the convolutional pose machine algorithm [60]. For clonic seizure detection, studies conducted by Ferrari et al. [30] and Pisani et al. [31] both focus on the periodicity of the motion signal which is quantified by background subtraction in video sequences, obtaining sensitivity and specificity above 90%. Martin et al. [32] acquired the velocity signal in videos using optical flow, extracting the average power spectral density of this signal to detect actual seizure events, significantly reducing the false-positive alarms by caregiver patting. Motion patterns are also related to pain and/or discomfort in infants. Sun et al. [28] extracted several features from the estimated motion acceleration rate signal derived from the velocity matrix of optical flow in the video sequences, achieving an AUC of 0.94 for the discomfort detection of infants in the NICU when compared to manual annotation. In their follow-up study, they took two motion matrices containing motion magnitudes of horizontal and vertical direction (derived from motion signal based on optical flow) as additional two channels combined with RGB channels to feed the classification model, achieving an AUC of 0.99 for discomfort detection [29]. To implement a multimodal analysis of infants’ pain, Zamzmi et al. [27] combined several scores for pain assessment including body motion, facial expression, and vital signs. In this study, the body motion was quantified by background subtraction of video sequences. Although the best performance was obtained by multimodality, the motion itself achieved an accuracy of 86%, recall of 55%, and precision of 52% on pain detection in infants.

Other applications that used motion quantification methods are infant presence detection and caregiver intervention detection. Long et al. [37] applied a block matching algorithm called 3D recursive search (3DRS) to quantify motion and detect the presence of infants in their beds, the similar idea was applied by Chabon [57] and Weber [61]. One step further, Chaichulee et al. [38] investigated a deep learning system for automatic skin segmentation from videos, simultaneously obtaining the presence and intervention status of the infants. In this study, the motion was quantified by optical flow and added together with skin confidence maps to detect intervention. The segmented skin without intervention is supposed to be used for following camera-based vital sign monitoring.

Camera-based technologies have shown promise for non-contact monitoring and motion pattern recognition, particularly in cases of motor impairments in infants. However, they still face several challenges. Firstly, with camera systems, the view of the infant during caregiving moments may be obstructed. Secondly, there may be privacy concerns associated with camera observation, although it is difficult to identify infants using IR cameras. Thirdly, due to the typically dim lighting in NICU environments and the potential covering of incubators, camera systems need to effectively handle low-light conditions. Detecting motion in such conditions presents a challenge, particularly for RGB cameras, emphasizing the enhanced utility of IR/thermal cameras. However, the latter has a significant drawback for neonatal applications, as the detection method fails if the incubator canopy is in between, and high-resolution IR/thermal cameras remain bulky and expensive and cannot easily be placed inside the incubator. Further investigations are necessary to enhance the effectiveness of camera-based sensing technologies.

#### 3.2.2 Radar-based

**Fig. 5:**
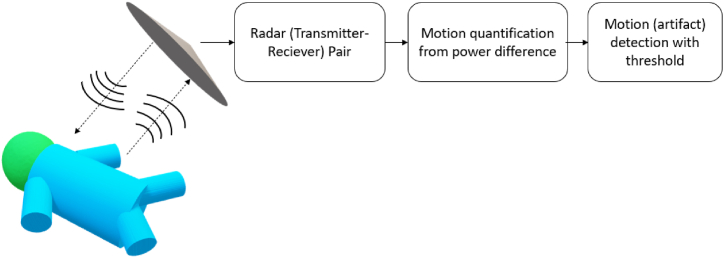
The block diagram of radar-based technology for motion detection.

Radar may be another promising non-contact monitoring technology in infants. Unlike the camera, radar can detect large and subtle motions (e.g. heart rate and respiration rate) regardless of light conditions and covering materials (such as clothing and blankets) [62]. The general block diagram of radar-based technology is shown in Fig. 5. In the context of infants, it has only been used so far to detect vital signs (motion of heart and/or chest) and has not been investigated for gross (or fine) body motion detection in itself, while large signal disturbances are considered motion artifacts. Lee et al. [40] used an impulse-radio ultra-wideband radar (IR-UWB) to monitor the cardiorespiratory signals in NICU. The motion of the infants was quantified with the power difference in radar signals. When the power difference was higher than a pre-defined threshold, the vital sign measurement became suspended due to the induced interference by body motion. In such a way, the estimation performance was only evaluated in an ideal scenario (no body motion) which is beyond the needs of clinical practice. Beltrao et al. [41] went one step further, instead of discarding the measurements during motion, they recovered the radar signal by quantifying and mitigating the body motion based on the time-frequency decomposition (Nonnegative matrix factorization or NMF), improving the estimation performance during motion and thereby being more in line with clinical practice.

Radar-based systems offer the advantage of detecting large and subtle motions regardless of light conditions. However, as highlighted in the review by Maurya et al. [2], certain limitations need to be addressed. Radar systems can be vulnerable to noise, making it challenging to detect 2D/3D movement patterns. Furthermore, the safety of using electromagnetic radiation with infants needs to be further investigated. Although it may be possible to detect gross motion using radar, distinguishing between patient movement and caregiver intervention can pose difficulties. Due to the limited research available on radar-based infant motion detection, its suitability is still uncertain.

#### 3.2.3 Mattress-based

**Fig. 6:**
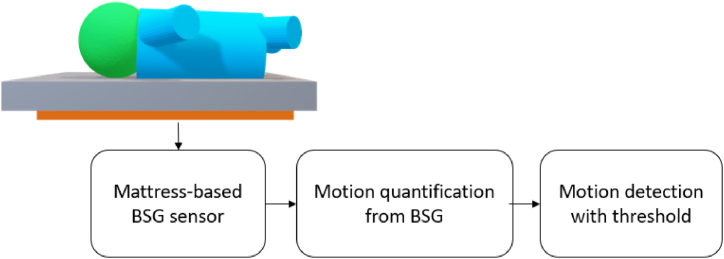
The block diagram of mattress-based technology for motion detection.

Ballistography (BSG) is a non-intrusive method of measuring the mechanical forces generated by the body, which can include forces due to body movement, breathing motion, and the mechanical action of the beating heart (also called ballistocardiography, BCG). Mattress-based sensing technologies utilize BSG sensors to measure the pressure generated by infants, including their body motion, chest motion, and heartbeats. Different types of BSG sensors and signal processing methods were investigated in monitoring infants using a mattress. Fig. 6 shows the block diagram of this technology for infant motion detection. For instance, Joshi et al. [42] conducted a motion detection study of 10 preterm infants using an electromechanical film sensor (EMFi) which is sensitive to forces on its surface [63]. Instead of directly using the BSG signal as the quantified motion signal, they first extracted several features (e.g. signal instability index and approximate entropy) with a 15-s sliding window from BSG, followed by comparing them with the video-based annotation to detect motion in infants. Besides BSG, these features were also extracted from electrocardiogram (ECG), chest impedance (CI), and photoplethysmogram (PPG) signals measured simultaneously for comparison. This study showed that the signal instability index from BSG outperformed other features from other signals with an AUC of 0.9 (0.06) on motion detection in infants. Additionally, Ranta et al. [44] used a piezoelectric film mattress sensor (L-4060SLC, Emfit) to record the BSG signals of 43 infants. The body motion was identified using simple thresholds applied to the smoothed root-meant-square value of the BSG signals. Afterwards, several features were extracted from this quantified motion signal to classify the sleep stage of infants, obtaining an AUC of 0.95. Due to the 1D output of these mattress sensors, only the magnitude but not the location of motion can be detected. A possible solution was reported by Aziz et al. [43] who used an LX100:100.100.05 pressure-sensitive mat (XSensor Technology Corp, Calgary) to measure BSG signals for motion detection. This sensor has a 2D output, allowing for the detection of limb motion. In this study, the motion of infants was quantified by the displacement of the center of pressure, and an AUC of 0.97 was achieved in motion detection in infants. The limb-specific motion was not evaluated in this study.

The utilization of BSG-based technologies is advantageous since they are non-obtrusive and can be positioned beneath a regular patient mattress, circumventing the risk of patient contact and infection. These technologies also enable the simultaneous measurement of body and chest motion alongside heartbeats, facilitating a comprehensive assessment of the infant's condition. However, caregiver interventions that are indistinguishable from patient motion may interfere with this technology, and the commonly used 1D output provides only limited information on motion patterns. Moreover, the sensitivity of the mattress is primarily restricted to motion perpendicular to it, with limited sensitivity to motion parallel to the mattress [64].

#### 3.2.4 Motion detection from clinically applied sensors

**Fig. 7:**
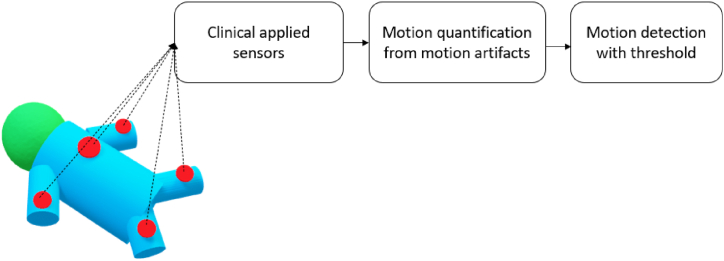
The block diagram of clinical sensor reusing technology for motion detection.

In clinical practice, patients’ vital signs are often monitored using ECG electrodes or PPG sensors. Motion can also be quantified from the signals of these sensors by first detecting motion artifacts in these signals and then quantifying the magnitude of the motion artifact as the motion signal as shown in Fig. 7.

Inspired by the work of Joshi et al. [42], Cabrera-Quiros et al. [48] quantified the instability of the monitored ECG signal as a motion signal by calculating the signal instability index from a sliding window in ECG. Specifically, the signal instability index was generated by kernel density estimation on ECG signals. As a motion artifact can affect the distribution of the raw ECG voltage traces, the bandwidth of the kernels increases in motion-affected periods. This bandwidth was termed the signal Instability Index to quantify the motion. In this study, the quantified motion was used to detect low-motion periods preceding sepsis in preterm infants.

Next to time domain components, properties in the frequency domain were also utilized to quantify motion as reported by Williamson et al. [45] and Zuzarte et al. [3]. They quantified the low-frequency component of the PPG signal as the motion signal because the motion artifact has stronger power in the low-frequency range. As a further step, they both extracted features from the motion signal to predict apnea events, proving the predictive value of motion signal in apnea prediction in infants [45,46]. Later, Peng et al. [47] compared and evaluated these artifact-based motion quantification methods (signal instability index, low-frequency power) and their combination of three physiological signals (ECG, CI, and PPG) with video-based annotation, showing the combined measure outperformed every single measure for motion detection in infants. Afterwards, this improved motion quantification method was utilized in a more recent study to highlight the complementary predictive value of motion information in relation to cardiorespiratory information in sepsis prediction in preterm infants [49].

One benefit of acquiring motion signals from sensors already attached to patients is that it imposes no extra burden on them and does not require additional time from caregivers. Additionally, these motion signals can serve as artifact detectors, improving the accuracy of clinical vital signs monitoring. However, the signal processing algorithms embedded in sensors for ECG and PPG typically include steps to decrease the sensitivity to motion, which can limit their effectiveness for detecting fine motion. Access to raw signals is necessary to achieve better motion quantification performance. Furthermore, the location of the attached sensor is not optimal for detecting limb motion. In home-care scenarios where regular patient monitoring is not conducted, attaching sensors to the skin solely for motion monitoring purposes may be unnecessary.

## 4 Discussion

This paper presents a systematic review of sensing technologies for motion quantification in infants. The aim is to address the gap in continuous monitoring of the body motion of infants, which is only determined by discrete periodic clinical observations of caregivers. In this section, we will first discuss the clinical value of infant motion monitoring. Afterwards, we will compare different sensing technologies for infant motion quantification and discusses their pros and cons for different clinical applications. In the end, we will discuss future research perspectives and suggest specific needs in clinical practice that can be referred by clinical users for their implementation.

### 4.1 Clinical value of quantified motion monitoring in infants

The motion is most frequently quantified as a separate physiological signal [10,23], preparing for the further analysis of various motion-related pathological conditions in infants as shown in Fig. 2. One point worth mentioning is that the detection of periods of reduced motion (e.g. in sepsis detection [48,49]), periodicity or changes in the motion patterns over time (e.g. in seizure detection [[30], [31], [32]]), startles during apnea [45,46], and motion counts in sleep assessment [4,12,44] can usually be acquired from a sensing technology providing 1D output signal where specific features can be extracted (e.g. number of bursts in a certain period, frequency, etc.). This 1D motion signal can also work for pain and discomfort detection [[27], [28], [29]] and these specific motion patterns can be independently or combined with other vital signs for pathology detection in infants. However, the 1D motion signal lacks detailed spatial information, and thus more advanced multimodal approaches may be needed to adequately recognize the motion development itself [10,27]. For instance, more advanced processing and modeling methods such as 3D motion reconstruction [13] and machine/deep learning [25,26,65] are needed to deal with detailed spatial information of motion for neuromotor pathologies, where the variation of limb motion and postures need to be recognized and estimated. Additionally, for camera-based non-contact vital sign monitoring, automatic infant body segmentation, intervention recognition, and pose estimation are all necessary steps [26,38,66].

In this area, many studies have shown the success of diagnosis using quantified motion, particularly by combining it with other vital signs for disease detection. To better characterize the diagnostic information from quantified motion signals, various features based on domain knowledge have been extracted to detect specific pathological statuses such as seizures, cerebral palsy, and sepsis as aforementioned in Refs. [30,49]. However, more studies investigating the association between motion patterns and health conditions in infants are needed to enhance knowledge and insight in this domain, verification by more clinical trials is also needed before implementation in clinical practice. This could eventually lead to motion signals being an additional vital sign for monitoring in intensive care settings or ward settings for (preterm) infants. The application of quantified motion in infants has not yet reached its full potential. For instance, in addition to disease diagnosis, motion is also associated with sleep analysis. Due to the important role of sleep in the maturation of infants, the correct detection of sleep stages of infants (with motion signal) can make sure that the quiet sleep would not be disturbed by caregiving moments. Moreover, the motion pattern potentially can also be a biomarker of infant maturity as illustrated in Refs. [44,67]. At this moment, there is no golden standard to determine motion as a clinical marker in practice, thus hampering the development of automatic motion monitoring techniques. Particularly, insufficient information is available on motion patterns of normal maturation and how to recognize abnormal motion patterns in a standardized way. This gap indicates a great development space for infant motion monitoring and may be potentially filled by applying advanced data-driven models to uncover knowledge of motion patterns in infants.

Additionally, to make vital sign monitoring more robust, motion quantification is also applied as an artifact detector. This often appears in applications of unobtrusive vital sign estimation, where the signal needs to be filtered and amplified to estimate the vital signs, making it vulnerable to artifact or noise. For artifact detection, usually, a 1D motion signal with a simple threshold based on magnitude is sufficient [35,40]. However, since it greatly reduces the value of the application to just discard those interfered measurements, the challenge of motion artifacts during unobtrusive or non-contact vital sign monitoring in NICUs remains. An important need in current clinical practice is therefore a continuous system that can make reasonably accurate vital sign estimation in different scenarios including infant motion. A current compromised solution based on the reviewed papers can be using signal processing methods such as singular value decomposition [68] and nonnegative matrix factorization [41] to mitigate the effect of motion artifact when the strength of the interference is allowable. When the interference is so strong that the measurements become unreliable, the motion signal can be recorded and used to reduce the false alarms caused by the strong motion.

### 4.2 Sensing technologies for motion quantification

Regarding sensing technologies for motion quantification, Table 3 shows that camera-based motion quantification methods were explored the most, followed by methods that extract motion from already applied clinically used sensors, based on artifact detection. When multiple options can be considered as sensing technology, the choice is based on how well the technology and processing fit the set of requirements for the applications and the possible risks. In Table 4, for each type of sensor, the applicability to several requirements for motion detection in infants in a hospital environment is outlined. All methods discussed in this table are suitable for motion quantification, though for neuromotor pathology detection, more advanced methods are needed to obtain sufficient spatial information (e.g. various body and limb motions), for which not all sensors and not all processing methods are suitable (and are not designed to perform this task).

**Table 4:** Comparison based on the requirements in the hospital environment for different types of sensing technologies. The annotations (+/−/--) represent the intensity of the recommendation.

Requirement	IMU-based	Camera-based: RGB	Camera-based: IR/thermal	Mattress-based	Radar-based	Signal-reusing
Being able to detect 1D gross motion (general gross motion)	+	+	+	+	+	+
Being able to detect 2D/3D gross motion patterns (also determine limb motion)	+	+	+	–	- -	- -
Being able to detect fine motion patterns	+	+	+	+	–	–
Minimal disturbance to the patient	- -	+	+	+	+	–
Being able to function in low-light conditions	+	- -	+	+	+	+
Being able to function in caregiving moments	+	- -	- -	–	- -	–
Being able to function if the incubator/patient is covered	+	- -	- -	+	–	+
No risk of skin damage	- -	+	+	+	+	–
No electrical risk to the patient	–	+	+	–	+	–
No possible risk of electromagnetic radiation exposure	–	+	+	–	–	–
No compromise to patient privacy	+	- -	–	+	+	+

The robustness of the motion detection method should be high and it should work in all conditions. Camera-based technology is highly dependent on surroundings, e.g. RGB camera can only function in adequate light conditions, whereas in low-light conditions an infra-red thermography camera can be used, however, this type of camera needs stable environment temperature. Moreover, the occlusion of view and the issue of privacy are also concerns for camera-based technologies. In contrast, IMU-based/accelerometer, radar-based and mattress-based technologies are less affected by surroundings and free of privacy problems. In addition to this, adaptability to the current clinical setting is also an important factor when considering practical sensing technologies, i.e. how many adjustments to the clinical setting (environment, protocols) are needed. For instance, the thermal camera needs a specialized hole in the incubator to access the infant because it cannot look through the glass cover of the incubator; additional positioning systems are needed to place the camera or radar at an adequate distance from the patient for an adequate view. In this aspect, reusing already applied sensors to detect motion from artifacts in these clinically acquired signals is the most convenient choice, because it needs no extra hardware. However, this technology has inherent limitations when monitoring motion. It often fails to detect fine motion due to the placement of the sensor it derives its information from and the signals from these sensors usually are already processed in such a way that motion artifacts are minimized.

The risks of applying the sensing technology should be low and they are also addressed in Table 4. In many papers, camera systems are investigated for motion monitoring, because of their ease of use and their low disturbance to regular clinical practice, not inducing additional safety risks to the infants compared with other technologies. However, the risks of failing to detect the motion signal can occur more frequently in camera systems (obstruction of view on the patient). For the other sensing technologies mentioned for motion monitoring, the technology itself may pose risks to the infant. For instance, radar-based technology may have a negative health impact on infants due to electromagnetic radiation exposure [69]. Devices with electronic components like mattress-based sensing (e.g. piezoelectric sensor) may pose an electrical safety risk, which can be mitigated by including additional safety steps in the design and can be measured using electrical safety testing. IMU-based wearable technologies may introduce risks of infection and skin damage as they need to be attached to the skin, which may be particularly a concern for preterm infants in NICU because of their vulnerable skin.

### 4.3 Open challenges of sensing technologies

Motion sensing technologies face many technical challenges of noise or artifacts in applications of continuous monitoring in real clinical conditions where patients are in motion and caregiving activities occur. The limitations of these technologies vary depending on the specific usage, and for detailed information regarding these limitations, we refer to the respective individual papers where they are addressed. In this section, we will address the main limitations that are set up independently for each method and discuss some possible directions for solutions as found in some of the reviewed papers. In camera-based technology, the main artifact comes from the changes in the field of view that are not generated by the infant body (e.g. nursing care), and suggestions to overcome this include using an accurate tracking of the region of interest (ROI) to detect infant motion. A promising solution may be the automatic body part segmentation and intervention recognition using deep learning as demonstrated by Antink et al. [66] and Chaichulee et al. [38]. A particular limitation of the RGB camera is that it cannot measure in typical dark light conditions in the incubator, though more sensitive cameras are still being developed and could improve the performance. A solution may be using IR cameras, however positioning them inside incubators requires the IR cameras to be small and also to function in humid atmospheres, which may limit their long time use. In the mattress-based technology, the disturbances on the mattress (e.g. by parents and caregivers) can also influence the measurement and it can lead to large artifacts cluttering the motion pattern of interest. Although the tracking of ROI by the center of pressure in a 2D mattress can overcome this artifact [43], further investigation is needed to fully recognize the intervention period using the mattress. Besides, most mattress-based sensors suffer from baseline drift, which can be addressed through post-processing correction methods. When the objective is to detect body motion, it is essential to consider other types of motion, such as respiration and heartbeat-related motions, as artifacts. However, employing bandpass filters can effectively isolate and prioritize the desired signal for accurate detection [42]. For motion detection in already regularly applied clinical sensors, an independent channel without reduction of motion artifacts should be obtained from the monitoring systems to determine the particular motion feature, which can only be done in collaboration with the vendors of these monitoring systems. In addition, filtering and further signal-processing methods can be also helpful when quantifying motion using clinically applied sensors [47].

### 4.4 Future direction for infant motion monitoring in clinical practice

With the advancement of technology, a possible future direction for infant motion monitoring in clinical practice could be an advanced ‘all-in-one’ system. This system can not only measure motion, preferably in 3D patterns as illustrated by Jeong et al. [13], but also can simultaneously measure multiple standard vital signs (including heart rate, respiration rate, temperature, and blood oxygenation), preferably in a less obtrusive way, using skin-safe sensors as illustrated by Chung et al. [10]. More importantly, using advanced learning algorithms that combine information from multiple sensors may facilitate the early detection of many complications in infants [70]. These advanced technologies still need further clinical evaluation in a larger number of infants representing a broad range of gestational age and real clinical conditions before implementation in clinical practice. In addition, future research could focus on the development of automated algorithms to analyze and interpret the motion signals, as well as on the integration of motion signals with other physiological signals to provide more comprehensive and accurate information on the infants' clinical status. Before their mature, reusing already applied sensors and obtaining extra information (like motion signals) from them seems a convenient solution for motion monitoring if the focus is on the gross motion. The mattress is a good alternative in case of spatial information of motion is needed, because of its possible 2D output. The camera can provide added value for research and as an additional sensor in detecting complex neuromotor motion patterns. For the general ward or home setting, where no complex medical equipment such as ventilators, incubators, and patient monitoring systems are in place for vital sign monitoring, the camera systems are also good candidates.

## 5 Conclusion

Clinical patient monitoring systems currently do not continuously measure motion signals and no sensor or technology is yet commercially available for these systems to detect patient motion. One major cause for this is that the clinical value of motion signal still needs to be further addressed by more clinical evaluations. In this work, motion quantification methods using different types of sensing technologies in various possible clinical applications have been reviewed to report the known clinical value of motion quantification in infants care, and the future trend with insights is presented as well. Clinical equipment is generally risk-averse, namely only the motion monitoring systems that are safe and robust in this complex environment hold the potential to become routinely used. Based on this review, it seems like the skin-safe adhesive ‘all-in-one’ sensors can be the choice for continuous motion monitoring when the technology is mature. Other sensing technologies should be applied depending on the specific application and environment in practice. Ultimately, this review shows promising results of both 1D output and more advanced motion quantification methods in different clinical applications using a variety of sensing technologies, meanwhile revealing the spacious room for the development of motion monitoring in infants, including its quantification and interpretation. The confluence of the diagnostic value of the motion signal itself combined with its value as an artifact detector may promote the adoption of continuous motion monitoring in clinical practice.

## Author contribution statement

All authors listed have significantly contributed to the development and the writing of this article.

## Data availability statement

No data was used for the research described in the article.

No additional information is available for this paper.

## Declaration of competing interest

The authors declare that they have no known competing financial interests or personal relationships that could have appeared to influence the work reported in this paper.

## References

[1] Muroi C., Meier S., De Luca V., Mack D.J., Strässle C., Schwab P., Karlen W., Keller E. (2020). Automated false alarm reduction in a real-life intensive care setting using motion detection. Neurocritical Care.

[2] Maurya L., Kaur P., Chawla D., Mahapatra P. (2021). Non-contact breathing rate monitoring in newborns: a review. Comput. Biol. Med..

[3] Zuzarte I., Indic P., Sternad D., Paydarfar D. (2019). Quantifying movement in preterm infants using photoplethysmography. Ann. Biomed. Eng..

[4] Schoch S.F., Jenni O.G., Kohler M., Kurth S. (2019). Actimetry in infant sleep research: an approach to facilitate comparability. Sleep.

[5] Marcroft C., Khan A., Embleton N.D., Trenell M., Plötz T. (2015). Movement recognition technology as a method of assessing spontaneous general movements in high risk infants. Front. Neurol..

[6] Mahallei M., Rezaee M.A., Mehramuz B., Beheshtirooy S., Abdinia B. (2018). Clinical symptoms, laboratory, and microbial patterns of suspected neonatal sepsis cases in a children's referral hospital in northwestern Iran. Méd..

[7] Raghuram K., Orlandi S., Church P., Chau T., Uleryk E., Pechlivanoglou P., Shah V. (2021). Automated movement recognition to predict motor impairment in high-risk infants: a systematic review of diagnostic test accuracy and meta-analysis. Dev. Med. Child Neurol..

[8] Chen H., Xue M., Mei Z., Oetomo S.B., Chen W. (2016). A review of wearable sensor systems for monitoring body movements of neonates. Sensors.

[9] Zhu Z., Liu T., Li G., Li T., Inoue Y. (2015). Wearable sensor systems for infants. Sensors.

[10] Chung H.U., Rwei A.Y., Hourlier-Fargette A., Xu S., Lee K.H., Dunne E.C., Xie Z., Liu C., Carlini A., Kim D.H., Ryu D., Kulikova E., Cao J., Odland I.C., Fields K.B., Hopkins B., Banks A., Ogle C., Grande D., Bin Park J., Kim J., Irie M., Jang H., Lee J.H., Park Y., Kim J., Jo H.H., Hahm H., Avila R., Xu Y., Namkoong M., Kwak J.W., Suen E., Paulus M.A., Kim R.J., Parsons B.V., Human K.A., Kim S.S., Patel M., Reuther W., Kim H.S., Lee S.H., Leedle J.D., Yun Y., Rigali S., Son T., Jung I., Arafa H., Soundararajan V.R., Ollech A., Shukla A., Bradley A., Schau M., Rand C.M., Marsillio L.E., Harris Z.L., Huang Y., Hamvas A., Paller A.S., Weese-Mayer D.E., Lee J.Y., Rogers J.A. (2020). Skin-interfaced biosensors for advanced wireless physiological monitoring in neonatal and pediatric intensive-care units. Nat. Med..

[11] Antony Raj A., Preejith S.P., Raja V.S., Joseph J., Sivaprakasam M. (2018). Clinical validation of a wearable respiratory rate device for neonatal monitoring. Proc. Annu. Int. Conf. IEEE Eng. Med. Biol. Soc. EMBS. 2018-July.

[12] Lan H.Y., Yang L., Hsieh K.H., Yin T., Chang Y.C., Liaw J.J. (2018). Effects of a supportive care bundle on sleep variables of preterm infants during hospitalization. Res. Nurs. Health.

[13] Jeong H., Kwak S.S., Sohn S., Lee J.Y., Lee Y.J., O'Brien M.K., Park Y., Avila R., Kim J.T., Yoo J.Y., Irie M., Jang H., Ouyang W., Shawen N., Kang Y.J., Kim S.S., Tzavelis A., Lee K.H., Andersen R.A., Huang Y., Jayaraman A., Davis M.M., Shanley T., Wakschlag L.S., Krogh-Jespersen S., Xu S., Ryan S.W., Lieber R.L., Rogers J.A. (2021). Miniaturized wireless, skin-integrated sensor networks for quantifying full-body movement behaviors and vital signs in infants. Proc. Natl. Acad. Sci. U. S. A..

[14] Tamura T., Chen W. (2018).

[15] Chung H.U., Kim B.H., Lee J.Y., Lee J., Xie Z., Ibler E.M., Lee K.H., Banks A., Jeong J.Y., Kim J., Ogle C., Grande D., Yu Y., Jang H., Assem P., Ryu D., Kwak J.W., Namkoong M., Bin Park J., Lee Y., Kim D.H., Ryu A., Jeong J., You K., Ji B., Liu Z., Huo Q., Feng X., Deng Y., Xu Y., Jang K.I., Kim J., Zhang Y., Ghaffari R., Rand C.M., Schau M., Hamvas A., Weese-Mayer D.E., Huang Y., Lee S.M., Lee C.H., Shanbhag N.R., Paller A.S., Xu S., Rogers J.A. (2019). Binodal, wireless epidermal electronic systems with in-sensor analytics for neonatal intensive care. Science.

[16] Sadeh A., Acebo C., Seifer R., Aytur S., Carskadon M.A. (1995). Activity-based assessment of sleep-wake patterns during the 1st year of life. Infant Behav. Dev..

[17] Lettink A., Altenburg T.M., Arts J., van Hees V.T., Chinapaw M.J.M. (2022). Systematic review of accelerometer-based methods for 24-h physical behavior assessment in young children (0–5 years old). Int. J. Behav. Nutr. Phys. Activ..

[18] Yang S.C., Yang A., Chang Y.J. (2014). Validation of actiwatch for assessment of sleep-wake states in preterm infants. Asian Nurs. Res..

[19] Guyer C., Huber R., Fontijn J., Bucher H.U., Nicolai H., Werner H., Molinari L., Latal B., Jenni O.G., Yang S.C., Yang A., Chang Y.J. (2015). Very preterm infants show earlier emergence of 24-hour sleep-wake rhythms compared to term infants. Early Hum. Dev..

[20] August D.L., Ray R.A., Kandasamy Y., New K. (2020). Neonatal skin assessments and injuries: nomenclature, workplace culture and clinical opinions—method triangulation a qualitative study. J. Clin. Nurs..

[21] Ossmy O., Gilmore R.O., Adolph K.E. (2019). AutoViDev A computer-vision framework to enhance and accelerate research in human development. Adv. Comput. Vis..

[22] Zhao M., Dai Z., Huang W., Sun K. (2020). Dynamic detection of infants' video based on all-programmable SoC in NICU, 2020 IEEE 3rd. Int. Conf. Autom. Electron. Electr. Eng. AUTEEE.

[23] Peng Z., van de Sande D., Lorato I., Long X., Liang R.H., Andriessen P., Cottaar W., Stuijk S., van Pul C. (2022). A comparison of video-based methods for neonatal body motion detection. Annu. Int. Conf. IEEE Eng. Med. Biol. Soc. IEEE Eng. Med. Biol. Soc. Annu. Int. Conf..

[24] Mazzarella J., McNally M., Richie D., Chaudhari A.M.W., Buford J.A., Pan X., Heathcock J.C. (2020). 3d motion capture may detect spatiotemporal changes in pre-reaching upper extremity movements with and without a real-time constraint condition in infants with perinatal stroke and cerebral palsy: a longitudinal case series. Sensors.

[25] Malik G., Gulati I.K. (2020). Little motion, big results: using motion magnification to reveal subtle tremors in infants. CEUR Workshop Proc.

[26] Wu Q., Xu G., Wei F., Kuang J., Zhang X., Chen L., Zhang S. (2021). Automatically measure the quality of infants' spontaneous movement via videos to predict the risk of cerebral palsy. IEEE Trans. Instrum. Meas..

[27] Zamzmi G., Pai C.Y., Goldgof D., Kasturi R., Ashmeade T., Sun Y. (2016). An approach for automated multimodal analysis of infants' pain. Proc. - Int. Conf. Pattern Recognit..

[28] Sun Y., Kommers D., Wang W., Joshi R., Shan C., Tan T., Aarts R.M., Van Pul C., Andriessen P., De With P.H.N. (2019). Automatic and continuous discomfort detection for premature infants in a NICU using video-based motion analysis. Proc. Annu. Int. Conf. IEEE Eng. Med. Biol. Soc. EMBS..

[29] Sun Y., Hu J., Wang W., He M., De With P.H.N. (2021). Camera-based discomfort detection using multi-channel attention 3D-CNN for hospitalized infants, Quant. Imaging Med. Surg..

[30] Ferrari G., Kouamou G.M., Copioli C., Raheli R., Pisani F. (2010). Low-complexity image processing for real-time detection of neonatal clonic seizures, 2010 3rd. Int. Symp. Appl. Sci. Biomed. Commun. Technol. ISABEL 2010.

[31] Pisani F., Spagnoli C., Pavlidis E., Facini C., Kouamou Ntonfo G.M., Ferrari G., Raheli R. (2014). Real-time automated detection of clonic seizures in newborns. Clin. Neurophysiol..

[32] Martin J.R., Gabriel P.G., Gold J.J., Haas R., Davis S.L., Gonda D.D., Sharpe C., Wilson S.B., Nierenberg N.C., Scheuer M.L., Wang S.G. (2022). Optical flow estimation improves automated seizure detection in neonatal EEG. J. Clin. Neurophysiol..

[33] Mestha L.K., Kyal S., Xu B., Lewis L.E., Kumar V. (2014). Towards continuous monitoring of pulse rate in neonatal intensive care unit with a webcam, 2014 36th. Annu. Int. Conf. IEEE Eng. Med. Biol. Soc. EMBC 2014.

[34] Rossol S.L., Yang J.K. (2017). Non-contact video-based neonatal respiratory monitoring. Congenit. Hear. Dis. Pediatr. Adult Patients Anesth. Perioper. Manag..

[35] Lorato I., Stuijk S., Meftah M., Kommers D., Andriessen P., van Pul C., de Haan G. (2021). Towards continuous camera-based respiration monitoring in infants. Sensors.

[36] Lyra S., Groß-Weege I., Leonhardt S., Lüken M. (2022). Real-time respiration monitoring of neonates from thermography images using deep learning. Lect. Notes Comput. Sci. (Including Subser. Lect. Notes Artif. Intell. Lect. Notes Bioinformatics). 13231 LNCS.

[37] Long X., Van Der Sanden E., Prevoo Y., Ten Hoor L., Den Boer S., Gelissen J., Otte R., Zwartkruis-Pelgrim E. (2018). An efficient heuristic method for infant in/out of bed detection using video-derived motion estimates. Biomed. Phys. Eng. Express..

[38] Chaichulee S., Villarroel M., Jorge J.o., Arteta C., McCormick K., Zisserman A., Tarassenko L. (2019). Cardio-respiratory signal extraction from video camera data for continuous non-contact vital sign monitoring using deep learning. Physiol. Meas..

[39] Badke D'Andrea C., Kenley J.K., Montez D.F., Mirro A.E., Miller R.L., Earl E.A., Koller J.M., Sung S., Yacoub E., Elison J.T., Fair D.A., Dosenbach N.U.F., Rogers C.E., Smyser C.D., Greene D.J. (2022). Real-time motion monitoring improves functional MRI data quality in infants. Dev. Cogn. Neurosci..

[40] Lee W.H., Lee Y., Na J.Y., Kim S.H., Lee H.J., Lim Y.H., Cho S.H., Cho S.H., Park H.K. (2021). Feasibility of non-contact cardiorespiratory monitoring using impulse-radio ultra-wideband radar in the neonatal intensive care unit. PLoS One.

[41] Beltrão G., Stutz R., Hornberger F., Martins W.A., Tatarinov D., Alaee-Kerahroodi M., Lindner U., Stock L., Kaiser E., Goedicke-Fritz S., Schroeder U., Zemlin M., B.S.M. R (2022). Contactless radar-based breathing monitoring of premature infants in the neonatal intensive care unit. Sci. Rep..

[42] Joshi R., Bierling B.L., Long X., Weijers J., Feijs L., Van Pul C., Andriessen P. (2018). A ballistographic approach for continuous and non-obtrusive monitoring of movement in neonates. IEEE J. Transl. Eng. Heal. Med..

[43] Aziz S., Dosso Y.S., Nizami S., Greenwood K., Harrold J., Green J.R. (2020). Detection of neonatal patient motion using a pressure-sensitive mat. IEEE Med. Meas. Appl. MeMeA 2020 - Conf. Proc..

[44] Ranta J., Airaksinen M., Kirjavainen T., Vanhatalo S., Stevenson N.J. (2021). An open source classifier for bed mattress signal in infant sleep monitoring. Front. Neurosci..

[45] Williamson J.R., Bliss D.W., Browne D.W., Indic P., Bloch-Salisbury E., Paydarfar D. (2013). Individualized apnea prediction in preterm infants using cardio-respiratory and movement signals. 2013 IEEE Int. Conf. Body Sens. Networks, BSN 2013.

[46] Zuzarte I., Sternad D., Paydarfar D. (2021). Predicting apneic events in preterm infants using cardio-respiratory and movement features. Comput. Methods Programs Biomed..

[47] Peng Z., Lorato I., Long X., Liang R.-H., Kommers D., Andriessen P., Cottaar W., Stuijk S., van Pul C. (2021). Body motion detection in neonates based on motion artifacts in physiological signals from a clinical patient monitor. 2021 43rd Annu. Int. Conf. IEEE Eng. Med. Biol. Soc., IEEE.

[48] Cabrera-Quiros L., Kommers D., Wolvers M.K., Oosterwijk L., Arents N., van der Sluijs-Bens J., Cottaar E.J.E., Andriessen P., van Pul C. (2021). Prediction of late-onset sepsis in preterm infants using monitoring signals and machine learning. Crit. Care Explor..

[49] Peng Z., Varisco G., Long X., Liang R.H., Kommers D., Cottaar W., Andriessen P., van Pul C. (2022). A continuous late-onset sepsis prediction algorithm for preterm infants using multi-channel physiological signals from a patient monitor. IEEE J. Biomed. Heal. Informatics..

[50] Sreenu G., Saleem Durai M.A. (2019). Intelligent video surveillance: a review through deep learning techniques for crowd analysis. J. Big Data..

[51] Wang X. (2013). Intelligent multi-camera video surveillance: a review. Pattern Recogn. Lett..

[52] Zivkovic Z., Van Der Heijden F. (2006). Efficient adaptive density estimation per image pixel for the task of background subtraction. Pattern Recogn. Lett..

[53] Horn B.K.P., Schunck B.G. (1981). Determining optical flow. Artif. Intell..

[54] Farneb G. (2003). Scand. Conf. Image Anal..

[55] de Haan G., Biezen P.W.A.C., Huijgen H., Ojo O.A. (1993). True-motion estimation with 3-D recursive search block matching. IEEE Trans. Circ. Syst. Video Technol..

[56] Rublee E., Rabaud V., Konolige K., Bradski G. (2011). Proc. IEEE Int. Conf. Comput.

[57] Cabon S., Porée F., Simon A., Ugolin M., Rosec O., Carrault G., Pladys P. (2017). Motion estimation and characterization in premature newborns using long duration video recordings. Irbm.

[58] Bochkovskiy A., Wang C.-Y., Liao H.-Y.M. (2020). http://arxiv.org/abs/2004.10934.

[59] Kreiss S., Bertoni L., Alahi A. (2019). PifPaf: composite fields for human pose estimation. Proc. IEEE Comput. Soc. Conf. Comput. Vis. Pattern Recognit. 2019-June.

[60] Ramakrishna V., Munoz D., Hebert M., Andrew Bagnell J., Sheikh Y. (2014). Pose machines: articulated pose estimation via inference machines. Lect. Notes Comput. Sci. (Including Subser. Lect. Notes Artif. Intell. Lect. Notes Bioinformatics). 8690 LNCS.

[61] Weber R., Cabon S., Simon A., Poree F., Carrault G. (2021). Preterm newborn presence detection in incubator and open bed using deep transfer learning. IEEE J. Biomed. Heal. Informatics..

[62] Mercuri M., Lorato I.R., Liu Y.H., Wieringa F., Van Hoof C., Torfs T. (2019). Vital-sign monitoring and spatial tracking of multiple people using a contactless radar-based sensor. Nat. Electron..

[63] Rajala S., Lekkala J. (2010). PVDF and EMFi sensor materials - a comparative study. Procedia Eng..

[64] Joshi R., Bierling B.L., Long X., Weijers J., Feijs L., Van Pul C., Andriessen P. (2018). A ballistographic approach for continuous and non-obtrusive monitoring of movement in neonates. IEEE J. Transl. Eng. Heal. Med..

[65] Ihlen E.A.F., Støen R., Boswell L., de Regnier R.A., Fjørtoft T., Gaebler-Spira D., Labori C., Loennecken M.C., Msall M.E., Möinichen U.I., Peyton C., Schreiber M.D., Silberg I.E., Songstad N.T., Vågen R.T., Øberg G.K., Adde L. (2020). Machine learning of infant spontaneous movements for the early prediction of cerebral palsy: a multi-site cohort study. J. Clin. Med..

[66] Antink C.H., Ferreira J.C.M., Paul M., Lyra S., Heimann K., Karthik S., Joseph J., Jayaraman K., Orlikowsky T., Sivaprakasam M., Leonhardt S. (2020). Fast body part segmentation and tracking of neonatal video data using deep learning. Med. Biol. Eng. Comput..

[67] Belmonti V., Cecchi F., Coluccini M., Ghirri P., Grassi A., Sabatini A.M., Guzzetta A. (2020). Early Human Development Movement analysis in early infancy : towards a motion biomarker of age. Early Hum. Dev..

[68] Li J., Liu L., Zeng Z., Liu F. (2014). Advanced signal processing for vital sign extraction with applications in UWB radar detection of trapped victims in complex environments. IEEE J. Sel. Top. Appl. Earth Obs. Rem. Sens..

[69] Calvente I., Vázquez-Pérez A., Fernández M.F., Núñez M.I., Múñoz-Hoyos A. (2017). Radiofrequency exposure in the neonatal medium care unit. Environ. Res..

[70] Guo R., Fang Y., Wang Z., Libanori A., Xiao X., Wan D., Cui X., Sang S., Zhang W., Zhang H., Chen J. (2022). Deep learning assisted body area triboelectric hydrogel sensor network for infant care. Adv. Funct. Mater..

